# Supplementary material for: Real-World Outcome and Prognostic Factors in MDS Patients Treated with Azacitidine—A Retrospective Analysis
Source: Cancers (Basel). 2024 Mar 29;16(7):1333. doi: 10.3390/cancers16071333 (PMC11011162; doi:10.3390/cancers16071333)
Supplement: Supplementary file 1 [file cancers-16-01333-s001.zip › Supplementary Figure.pdf]

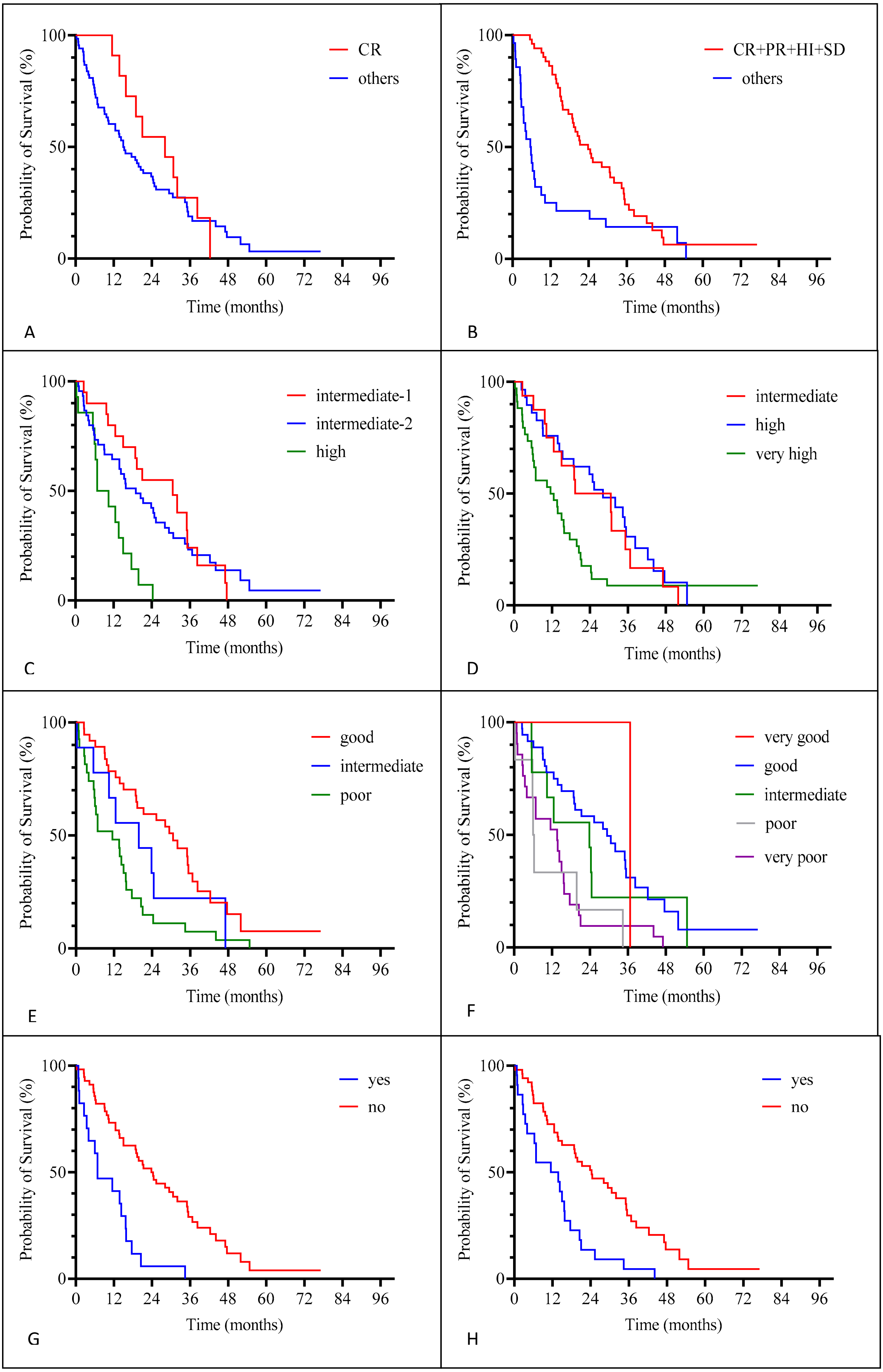

Probability of Survival (%)
Time (months)
0
50
100
0
12
24
36
48
60
72
84
96
A
CR
others
B
CR+PR+HI+SD
others
C
intermediate-1
intermediate-2
high
D
intermediate
high
very high
E
good
intermediate
poor
F
very good
good
intermediate
poor
very poor
G
yes
no
H
yes
no

Supplementary Figure S1 Prognostic factors of overall survival
A. Treatment response (CR vs others); B. Treatment response (CR+PR+HI+SD vs others); C. IPSS risk group; D. R-IPSS risk group; E. Cytogenetic risk (IPSS); F. Cytogenetic risk (IPSS-R); G. Complex karyotype; H. Monosomal karyotype.

CR: complete remission; PR: partial remission; HI: hematologic improvement; SD: stable disease; IPSS: International Prognostic Scoring System; IPSS-R: International Prognostic Scoring System revised
