# Supplementary material for: Real-World Outcome and Prognostic Factors in MDS Patients Treated with Azacitidine—A Retrospective Analysis
Source: Cancers (Basel). 2024 Mar 29;16(7):1333. doi: 10.3390/cancers16071333 (PMC11011162; doi:10.3390/cancers16071333)
Supplement: Supplementary file 1 [file cancers-16-01333-s001.zip › Supplementary Tables.pdf]

Supplementary Table S1
Incidence rate of infectious complications.

Number of infection episodes, n	Number of patients, n (%)
**0**	24 (30.4%)
**1**	22 (27.9%)
**2-3**	24 (30.4%)
**4**	6 (7.6%)
**≥ 5**	3 (3.8%)

Supplementary Table S2
Schedule modifications and dose adjustment in AZA treatment.

Type of intervention	Number of patients, n (%)
**Dose reduction**	
Yes	23 (29,1%)
No	56 (70.9%)
**Cycle reduction (< 7 days)**	
Yes	25 (31,6%)
No	54 (68.4%)
**Treatment delay**	
Yes	7 (8,9%)
No	72 (91.1%)

Supplementary Table S3
Univariate analysis of factors influencing response to treatment.

Parameter Median (range)	Treatment response		p
	CR + PR + HI + SD n=51	PD n=28	
**Bone marrow blasts**	8 (0.8-19)	10 (0.6-15.8)	0.69
**WBC** (G/l)	2.3 (0.6-11.5)	2.2 (0.1-17.8)	0.99
**ANC** (G/l)	0.9 (0.0-7.9)	0.9 (0.1-10.8)	0.68
**HGB** (g/dl)	7.9 (4.2-13.8)	7.8 (5.4-10.4)	0.27
**PLT** (G/l)	64 (10-343)	53.5 (7-300)	0.22
**LDH** (U/l)	393 (162-1064)	415 (220-1426)	0.99
**Serum ferritin** (ng/ml)	403 (41-6552)	656.7 (12.3-10410)	0.39
**Age** (years)	69 (42-89)	68.5 (59-85)	0.49
**BMI**	25.7 (18-36.1)	26.6 (19.3-36.1.)	0.49
**HCT-CI**	1 (0-6)	1 (0-7)	0.55

WBC: white blood cell; ANC: absolute neutrophil count; HGB: hemoglobin; PLT: platelets; LDH: lactate dehydrogenase; BMI: body mass index; HCT-CI: The Hematopoietic cell transplantation-specific comorbidity index; ECOG: Eastern Cooperative Oncology Group.

Supplementary Table S4
Univariate analysis of factors associated with treatment response.

Parameter	Treatment response				p
	CR + PR + Hi + SD n=51		PD n=28		
	n	%	n	%	
**Gender**					
Male	27	60.0	18	40.0	0.33
Female	24	70.6	10	29.4	
**WHO diagnosis**					
MDS-MLD	5	83.3	1	16.7	0.79
MDS-RS	1	100.0	0	0.0	
MDS EB-1	17	65.4	9	34.6	
MDS-EB-2	24	60.0	16	40.0	
MDS 5q-	1	100.0	0	0.0	

t-MDS	3	60.0	2	40,0	
**IPSS risk group**					
Intermediate-1	16	80.0	4	20.0	0.24
Intermediate-2	27	60.0	18	40.0	
High	8	57.1	6	42.9	
**IPSS-R risk group**					
Intermediate-2	13	81.3	3	18.8	0.12
High	20	69.0	9	31.0	
Very high	18	52.9	16	47.1	
**Cytogenetic risk (IPSS)**					
Good	27	73.0	10	27.0	**0.03**
Intermediate	8	88.9	1	11.1	
Poor	13	48.1	14	51.9	
**Cytogenetic risk (IPSS-R)**					
Very good	1	100.0	0	0	
Good	26	72.2	10	27.8	0.39
Intermediate	6	66.7	3	33.3	
Poor	2	33.3	4	66.7	
Very poor	13	61.9	8	38.1	
**Complex karyotype**					
Yes	13	59.1	9	40.9	0.43
No	35	68.6	16	31.4	
**Monosomal karyotype**					
Yes	9	52.9	8	47.0	0.2
No	39	69.6	17	30.4	
**Peripheral blood blasts**					
Present	18	64.3	10	35.7	0.84
Absent	28	66.7	14	33.3	
**BMI**					
18,50 <	1	100.00	0	0.0	
18,50-24,99	19	67.9	9	32.1.	0.63
25-29,99	23	63.9	13	36.1.	
> 30	8	61.5	5	38.5	
**Transfusion dependence**					
RBC-TD	26	61.9	16	38.1	0.19
RBC-TD + PLT-TD	7	50.0	7	50.0	
Transfusion independent	18	78.3	5	21.7	

CR: complete remission; PR: partial remission; HI: hematologic improvement; SD: stable disease; PD: progressive disease; WHO: World Health Organization; MDS: myelodysplastic syndrome; MDS-MLD: myelodysplastic syndrome with multilineage dysplasia; MDS-RS: myelodysplastic syndrome with ring sideroblasts; MDS-EB: myelodysplastic syndrome with excess of blasts; U: unclassifiable; t-MDS: therapy-related myelodysplastic syndrome; IPSS: International Prognostic Scoring System; IPSS-R: International Prognostic Scoring System revised; BMI: body mass index; RBC: red blood cell; TD: transfusion dependent; PLT: platelets.
